# A Kinase Interacting Protein 1 regulates mitochondrial protein levels in energy metabolism and promotes mitochondrial turnover after exercise

**DOI:** 10.1038/s41598-023-45961-z

**Published:** 2023-11-01

**Authors:** Kirsten T. Nijholt, Pablo I. Sánchez-Aguilera, Belend Mahmoud, Albert Gerding, Justina C. Wolters, Anouk H. G. Wolters, Ben N. G. Giepmans, Herman H. W. Silljé, Rudolf A. de Boer, Barbara M. Bakker, B. Daan Westenbrink

**Affiliations:** 1 Department of Cardiology, University Medical Centre Groningen, University of Groningen, Hanzeplein 1, 9713 GZ Groningen, The Netherlands; 2 Department of Metabolic Disease, University Medical Centre Groningen, University of Groningen, Groningen, The Netherlands; 3 Department of Pediatrics, Systems Medicine of Metabolism and Signalling, University Medical Centre Groningen, University of Groningen, Groningen, The Netherlands; 4 Department of Biomedical Sciences of Cells and Systems, University Medical Centre Groningen, University of Groningen, Groningen, The Netherlands; 5 Department of Cardiology, Erasmus University Medical, Rotterdam, The Netherlands

**Keywords:** Cardiovascular biology, Metabolism, Mitochondria, Cardiac hypertrophy, Molecular biology, Molecular medicine

## Abstract

A Kinase Interacting Protein 1 (AKIP1) is a signalling adaptor that promotes mitochondrial respiration and attenuates mitochondrial oxidative stress in cultured cardiomyocytes. We sought to determine whether AKIP1 influences mitochondrial function and the mitochondrial adaptation in response to exercise in vivo. We assessed mitochondrial respiratory capacity, as well as electron microscopy and mitochondrial targeted-proteomics in hearts from mice with cardiomyocyte-specific overexpression of AKIP1 (AKIP1-TG) and their wild type (WT) littermates. These parameters were also assessed after four weeks of voluntary wheel running. In contrast to our previous in vitro study, respiratory capacity measured as state 3 respiration on palmitoyl carnitine was significantly lower in AKIP1-TG compared to WT mice, whereas state 3 respiration on pyruvate remained unaltered. Similar findings were observed for maximal respiration, after addition of FCCP. Mitochondrial DNA damage and oxidative stress markers were not elevated in AKIP1-TG mice and gross mitochondrial morphology was similar. Mitochondrial targeted-proteomics did reveal reductions in mitochondrial proteins involved in energy metabolism. Exercise performance was comparable between genotypes, whereas exercise-induced cardiac hypertrophy was significantly increased in AKIP1-TG mice. After exercise, mitochondrial state 3 respiration on pyruvate substrates was significantly lower in AKIP1-TG compared with WT mice, while respiration on palmitoyl carnitine was not further decreased. This was associated with increased mitochondrial fission on electron microscopy, and the activation of pathways associated with mitochondrial fission and mitophagy. This study suggests that AKIP1 regulates the mitochondrial proteome involved in energy metabolism and promotes mitochondrial turnover after exercise. Future studies are required to unravel the mechanistic underpinnings and whether the mitochondrial changes are required for the AKIP1-induced physiological cardiac growth.

## Introduction

One of the central mechanisms in heart failure (HF) is the development of pathological cardiac hypertrophy^1,2^. This maladaptive growth response is often accompanied by alterations in mitochondrial morphology, excessive reactive oxygen species (ROS) formation, alterations in mitochondrial calcium (Ca^2+^) handling, disruptions in mitochondrial dynamics leading to mitochondrial dysfunction and ultimately in lower myocardial adenosine triphosphate (ATP) production^1,2^.

Cardiac hypertrophy is, however, not inherently maladaptive as it also occurs in response to physiological stressors such as pregnancy and exercise^1–5^. In contrast to pathological cardiac hypertrophy, this type of cardiac growth is often associated with improvements in metabolism. For instance, exercise promotes mitochondrial quality control by stimulating mitochondrial turnover, decreases mitochondrial ROS production, stimulates mitochondrial Ca^2+^ handling and improves mitochondrial ATP production^1–5^. Together, these exercise-induced mitochondrial changes are thought to be adaptive and mitochondrial function is maintained or improved^1–4^.

With the knowledge that exercise exerts beneficial cardiac mitochondrial and cardiac growth effects in health and disease^1,4^, novel targets for treatment of HF may be discovered by improving our understanding of the mechanistic pathways underlying these mitochondrial adaptations. Identifying nodal points in the regulation of these adaptive responses may lead to therapies that would allow us to switch mitochondrial responses from a pathological to a more physiological phenotype.

A Kinase Interacting Protein 1 (AKIP1) has recently been identified as an important signalling adaptor in the heart and promotes a physiological type of cardiac growth in cultured cardiomyocytes and in vivo murine hearts through Akt signalling^6,7^. The role of AKIP1 in response to pathological stress was limited for cardiac function and pathological hypertrophy in cardiomyocyte-specific AKIP1 transgenic (AKIP1-TG) mice^8^, but AKIP1 did protect from ischemia/reperfusion (I/R) injury through mitoprotection^8,9^. In these two studies, AKIP1 was also observed to localize to the mitochondria^8,9^. Studies with neonatal rat ventricular cardiomyocytes (NRVM) also suggest a role for AKIP1 in mitochondria as mitochondrial respiration was improved with AKIP1 overexpression^10^. This effect was accompanied by increments in ATP production rate and decrements in ROS production^10^. It remains unknown whether AKIP1, as a kinase, regulates mitochondrial function and the cardiac mitochondrial adaptation to exercise in vivo. It also remains unknown whether the metabolic substrate utilization with AKIP1 mimics a more physiological or pathological phenotype. Therefore, we aimed to assess mitochondrial function, using glucose and fatty acid substrates, as well as the mitochondrial response to physiological stress in a cardiomyocyte-specific AKIP1 transgenic mouse model.

## Methods

### Ethical approval

Ethical approval for the animal experiments was provided by the Animal Ethics Committee from the University of Groningen (IvD 199105-01-005). The animal experiments were performed in accordance with the protocols from Directive 2010/63/EU of the European Parliament and in accordance with the ARRIVE guidelines.

### Animal model

This study included the use of a transgenic mouse model in which there was cardiomyocyte-specific overexpression of A Kinase Interacting Protein 1 (AKIP1-TG), as described previously^8^. mRNA expression of AKIP1 was determined to validate AKIP1 overexpression (Supplementary Fig. S1). Wild type (WT) mice from the same breeding line were used as controls. The total study population included an N = 58, with male mice aged 8–12 weeks old at the start of the experiment. For this experiment we wanted to assess our primary and secondary outcomes in adult male mice, we therefore selected the age range of 8–12 weeks old at the start of the experiment. With a total duration of 4 weeks for the experiment to be completed, analyses were performed on tissue at 12–16-week-old mice.

### Experimental model

AKIP1-TG and WT mice were housed individually for a period of four weeks, with or without the presence of a running wheel available in their cage. Mice that were subjected to four weeks of voluntary wheel running will be referred to as WT Run and AKIP1-TG Run, whereas sedentary mice will be referred to as WT Sed and AKIP1-TG Sed.

### Exercise performance

To determine exercise performance, running parameters including running distance, speed and time were measured using a cyclometer and were recorded daily^11^.

### Organ and body measurements

After the four-week experimental period, mice were anesthetized with 2% isoflurane and euthanized by cardiac puncture, which was followed by blood draw, flushing with saline, and excision and weighing of the heart. For mitochondrial function assays, the heart was further processed for mitochondrial isolation on the same day of sacrifice. For cardiac molecular analysis, the heart was separated into atria, right ventricle (RV) and left ventricle (LV) and were snap frozen in liquid nitrogen.

### Mitochondrial isolation

Mitochondrial isolation was done directly after sacrifice and performed for different purposes, including determination of mitochondrial respiratory capacity, citrate synthase activity assay and mitochondrial-targeted proteomics. Mitochondria were isolated as previously described^12^. In brief, hearts were kept on ice in 0.9% KCL directly after sacrifice and cut into smaller pieces in medium A (220 mM mannitol, 70 mM sucrose, 5 mM TES, 0.1 mM EGTA, pH 7.3 at 4 °C with 1N KOH) with added proteinase (P8038, Sigma, USA) for five minutes. This was followed by adding medium A supplemented with bovine serum albumin and homogenizing the samples using a Potter–Elvehjem homogenizer. 500 µl of this homogenate was stored as a homogenate sample. Thereafter, samples were centrifuged three times with the result of a mitochondrial pellet, which was resuspended in medium A and remained on ice.

### Mitochondrial protein content quantification

After mitochondrial isolation, mitochondrial protein content was quantified using a BCA protein assay (Pierce. No. 232250, ThermoFisher, USA). Different dilutions were used to increase accuracy of measurements: 2×, 4×, and 16× dilutions were made for mitochondrial samples and 2×, 4×, and 8× dilutions were made for homogenate samples.

### Mitochondrial respiratory capacity

Respirometry of isolated cardiac mitochondria was measured directly after mitochondrial isolation using a two-channel high-resolution Oroboros oxygraph-2 k (Oroboros, Innsbruck, Austria), which could detect changes in oxygen (O_2_) fluxes at 37 °C, as described before^12^. Prior to measurements, the two channels were washed and calibrated by adding 1 ml of the assay medium MiR05 (110 mM sucrose, 60 mM potassium lactobionate, 20 mM taurine, 20 mM HEPES, 0.5 mM EGTA, 10 mM KH_2_PO_4_, 3 mM MgCl_2_, 1 mg/ml bovine serum albumin, at pH 7.1). Next, 10 µl of mitochondria was added and state 2 respiration was measured. This was followed by adding 2 mM pyruvate and 2 mM malate to the system when assessing glucose-derived substrate utilization, and when assessing fatty acid utilization, 25 µM palmitoyl carnitine and 2 mM malate were added. Thereafter, for both glucose and fatty acid protocols, 10 mM glucose, 1.5 U/ml hexokinase and 1 mM ATP was added to induce state 3 respiration. State 4 respiration was measured by adding 1.25 µM carboxyatractyloside and state uncoupled by adding 1.5 µM FCCP. 10% sodium dithionite was added to remove all oxygen in the channel. Data of O_2_ fluxes were collected and analysed in DatLab (Version 5.1, Oroboros, Innsbruck, Austria), normalized for the total mitochondrial protein content and presented in nmol/min/mg mitochondrial protein.

### Citrate synthase activity assay

Both a homogenate and a mitochondrial sample were stored for determining citrate synthase activity assay as a measure for mitochondrial mass, which has been reported previously^12^. This entails a spectrophotometric assay in which the production of thionitrobenzoic acid (TNB) is measured at a wavelength of 412 nm at 37 °C. The enzymatic reaction involving citrate synthase is as follows: acetyl-CoA, oxalacetate and H_2_O to form citrate and coenzyme A (CoA). In the presence of dithionitrobenzoic acid (DTNB), CoA binds to DTNB to form TNB and an increase in TNB absorbance can be measured while the enzymatic reaction takes place. The reagent mixture for the assay contained: H_2_O, 10% Triton X-100, 10 mM oxaloacetate and 1 mM DTNB. The enzymatic reaction was initiated by addition of 12.2 mM acetyl-CoA. Citrate synthase activity was presented in nmol/min/mg and was normalized for the protein content measured in the initial sample, which was either homogenate or mitochondrial.

### Mitochondrial targeted proteomics

To quantify a broad spectrum of mitochondrial proteins involved in energy metabolism, mitochondrial targeted proteomics was performed, comprehensively targeting proteins involved in fatty acid β-oxidation, tricarboxylic acid cycle (TCA), oxidative phosphorylation (OXPHOS), substrate transport and antioxidant activity, The protocol used was according to previous publications by Wolters et al.^13^ and Stolle et al.^12^, which included the use of isotopically labelled standards (^13^C-labeled lysines and arginines) obtained from synthetic protein concatemers (QconCAT) (PolyQuant GmbH, Bad Abbach, Germany)*.* In short, 30 µg mitochondrial protein was used to prepare samples according to an in-gel trypsin digestion protocol. Preparation of samples included addition of 1.5 ng QconCAT per 1 µg of mitochondrial protein, reduction with 10 mM dithiothreitol, alkylation with 55 mM iodoacetamide, in-gel tryptic digestion (1:100 g/g) and finally peptide extraction. After preparation, samples were analysed using a triple quadrupole mass spectrometer (MS) with a nano-electrospray ion source (TSQ Vantage, Thermo Scientific, USA). Liquid chromatography on a nano-UHPLC system (Ultimate UHPLC focused, Dionex) was used to separate peptides (gradient 100 min). Tracing of MS peaks was performed manually using Skyline software, whereafter quantification of peak areas was done. Endogenous peptide levels were normalized for the levels of QconCAT peptide standards and final mitochondrial protein levels were presented in fmol/µg total mitochondrial protein.

### Electron microscopy

To evaluate mitochondrial mass and morphology, large-scale electron microscopy (EM), also referred to as nanotomy and was performed as described before^14,15^*.* Directly following sacrifice, cardiac tissue from the mid-papillary level was cut into small pieces of approximately 1 mm^2^ and fixated in 2% glutaraldehyde and 2% paraformaldehyde solution in 0.1 M sodium cacodylate. Next, samples were postfixed with 1% osmiumtetraoxide and 1.5% potassium ferrocyanide. After dehydration, samples were embedded in EPON epoxy resin and with an ultramicrotome 80 nm sections were cut. Contrast staining was then performed with 4% neodymium acetate and finally samples were imaged using scanning and transmission electron microscopy (STEM) (Zeiss, Supra55, Oberkochen, Germany). A ‘nanotomy’ map was generated by an external scan generator ATLAS5 (Fibics, Canada) and TIFF files were exported to html files. Raw data of html files can be viewed at www.nanotomy.org. For analysis of mitochondria and morphology, six high-resolution images were selected at random from each nanotomy file and images were analysed in Fiji-ImageJ software (Fiji-ImageJ version of Java 6, USA).

### Quantitative real-time polymerase chain reaction (qRT-PCR)

To assess mitochondrial molecular markers at mRNA level we performed qRT-PCR, as described before^16^*.* Total RNA was isolated from snap frozen and powdered LV tissue using Trizol reagent (Invitrogen Corporation, USA). After RNA quantification and purity check using Nanodrop (ThermoFisher, USA), 500 ng of RNA was reverse transcribed to cDNA with Quantitect Reverse Transcription kit (Qiagen, the Netherlands, no. 205313). Amplification of cDNA occurred with qRT-PCR using different sets of primers as listed in Supplementary Table S1. The following running protocol was used: 3 min at 95 °C, followed by 35 cycles of (1) 15 s at 95 °C, (2) 30 s at 60 °C; followed by a dissociation step and melting steps (Bio-Rad CFX384, USA). Data were processed using the ddCT method and normalized for both housekeeping gene 36b4 and wild type control group.

### Mitochondrial DNA to nuclear DNA ratio (mitochondrial DNA copy number)

As a measure for mitochondrial biogenesis and content, mitochondrial DNA copy number was determined by qRT-PCR using primers to determine the ratio between mitochondrial and nuclear DNA (mtDNA and nDNA respectively)^16^*.* Total DNA was isolated from snap frozen and powdered LV tissue using the Nucleospin Tissue XS kit (Macherey–Nagel, Germany). After quantification of DNA concentrations using the Nanodrop (ThermoFisher, USA), 10 µg were used for qRT-PCR using a similar running protocol as mentioned above. The primers used for mtDNA were for the mtDNA-encoded protein NADH dehydrogenase (ND1) and the primers used for the nDNA were for the nDNA-encoded hexokinase 2 (HK2) and primer sequences are included in Supplementary Table S1. Data were processed using the ddCT method and normalized for both reference gene HK2 and wild type control group to determine mtDNA copy number.

### Mitochondrial DNA damage

Mitochondrial DNA damage was determined to detect lesions in mitochondrial DNA, as previously reported^17^. Total DNA was isolated and quantified as described above, and again 10 µg of DNA was used for qRT-PCR. Two different protocols were run to determine mtDNA short and long fragments, which its ratio calculates for the damage lesions in mtDNA (primer sequences are included in Supplementary Table S1). The protocol for mtDNA short fragment used was 95 °C for 10 min, followed by 40 cycles of 10 s at 95 °C, 10 s at 60 °C, and 10 s 72 °C. The protocol for mtDNA long fragment used was 95 °C for 10 min, followed by 40 cycles of 10 s at 95 °C, 10 s at 60 °C, and 50 s 72 °C. Data were calculated according to the ddCT method, mtDNA short fragment served as reference gene and data were normalized for the wild type control group.

### Western blot

Western blots were performed to assess mitochondrial molecular markers at protein level, as previously reported^16^*.* Total protein was isolated from snap frozen and powdered LV tissue using radioimmunoprecipitation assay (RIPA) buffer, fresh sodium vanadate, phosphatases, and proteases inhibitors (Sigma-Aldrisch, USA). Protein quantifications were determined by using the BCA protein assay (Pierce. No. 232250, ThermoFisher, USA). Equal quantities of protein samples were prepared with RIPA buffer and 5× loading buffer and boiled at 99 °C for 5 min. Whereafter samples were loaded onto sodium dodecyl sulphate–polyacrylamide gel for electrophoresis (SDS-PAGE). After completion of electrophoresis, proteins were transferred onto polyvinylidene difluoride (PVDF) membranes by semi-dry blotting. To assess multiple proteins of interest from the same blot and same samples, some membranes were cut at this stage. After blocking, membranes were incubated overnight at 4 °C with primary antibodies, as listed in Supplementary Table S2. After 1-h incubation with secondary antibodies the next day, membranes were detected with enhanced chemiluminescence solution (ECL) (Pierce, ThermoFisher, USA) in ImageQuant imager. Acquired images were analysed and quantified using Fiji-ImageJ software (Fiji-ImageJ version of Java 6, USA). Quantifications were normalized for loading controls GAPDH or total protein stain and for the wild type control group. For the main text figures, western blots were cropped. The original images are provided in Supplementary Information File 1.

### Statistical analysis

Data are presented using mean and standard error of the mean (SEM). Statistical significance was considered with a p value < 0.05. Distribution of data was determined by a normality test. Comparison between two groups with normally distributed data was done with a Student’s *t*-test and for comparison with not normally distributed data a Mann–Whitney U test was used. For statistical comparison between more than two groups, a two-way ANOVA was performed. As described before^8^, this allowed for assessment of interactive effects between genotype and exercise variables. If statistical significance was observed, further multiple comparisons between all groups with post hoc Tukey’s correction test was performed. Statistical analysis was performed using GraphPad prism software (Version 7, USA).

## Results

### AKIP1 decreases mitochondrial state 3 respiration for palmitoyl carnitine but not for pyruvate, while state 4 respiration remains preserved for both substrates

**Figure 1:**
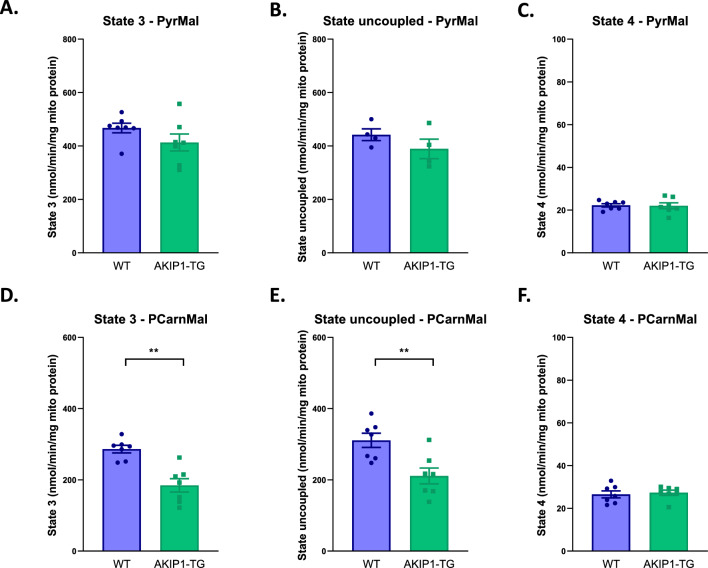
Mitochondrial function and integrity: mitochondrial state 3, state uncoupled and state 4 respiratory capacity. Shown are (**A**) state 3 respiration with pyruvate and malate (PyrMal) in nanomole per minute per milligram of mitochondrial protein (nmol/min/mg mito protein) (N = 7/group), (**B**) state uncoupled respiration with PyrMal in nmol/min/mg mito protein (N = 4–7/group), (**C**) state 4 respiration for PyrMal in nmol/min/mg mito protein (N = 7/group), (**D**) state 3 respiration for palmitoyl carnitine and malate (PCarnMal) substrates in nmol/min/mg mito protein (N = 7/group), (**E**) state uncoupled respiration with PCarnMal in nmol/min/mg mito protein (N = 7/group), (**F**) state 4 respiration for PCarnMal in nmol/min/mg mito protein (N = 7/group). WT, wild type mice; AKIP1-TG, AKIP1 transgenic mice. Graphs represent mean ± standard error of the mean (SEM). Statistical analysis for comparing two groups was performed with Student’s *t-*test or Mann–Whitney U test. < 0.05 was considered statistically significant; p** < 0.01.

We initially aimed to determine mitochondrial respiratory capacity as a measure for mitochondrial function in AKIP1-TG mice (typical examples of respirometry curves measured by the two-channel high-resolution Oroboros oxygraph-2 k system can be found in Supplementary Fig. S2). First, we measured mitochondrial state 3 respiration on a glucose-derived substrate (pyruvate), as well as on a fatty acid-derived substrate (palmitoyl carnitine). State 3 respiration on pyruvate did not differ between sedentary AKIP1-TG compared to WT mice (Fig. 1A). Similar findings were observed in the state uncoupled respiration state after addition of FCCP, in both sedentary WT and AKIP1-TG mice (Fig. 1B). Additionally, state 4 respiration remained unchanged in both groups (Fig. 1C). Contrary to this, state 3 respiration with a fatty-acid derived substrate (palmitoyl carnitine) was significantly diminished in AKIP1-TG compared to WT mice (Fig. 1D), as was state uncoupled respiration after FCCP addition (Fig. 1E). AKIP1 did however not influence the state 4 respiration (Fig. 1F), suggesting that the mitochondrial membrane remained intact despite reductions in state 3 respiration.

### Mitochondria in AKIP1-TG mice do not appear to be damaged despite decreased mitochondrial function

To better understand whether the observed changes in mitochondrial function were associated with defects in mitochondrial integrity and cardiac oxidative stress, we evaluated molecular markers for mitochondrial damage and oxidative stress. We first assessed mitochondrial DNA damage, which did not differ between the two groups (Supplementary Fig. S3). Next, we examined cardiac mRNA expression of markers for oxidative stress, including nuclear respiratory factor 2 (NRF2) and NADPH oxidase 2 (NOX2), which were comparable between WT and AKIP1-TG mice (Supplementary Fig. S3). Additionally, protein levels of antioxidant enzymes superoxide dismutase 2 (SOD2) and glutathione peroxidase 4 (GPX4) were not significantly different between AKIP1-TG and WT mice (Supplementary Information File 2). These findings suggest that the reductions in mitochondrial respiration are not caused by enhanced mitochondrial damage or excessive oxidative stress in AKIP1-TG mice.

### Mitochondrial morphology, architecture and abundance are not affected by AKIP1 overexpression

**Figure 2:**
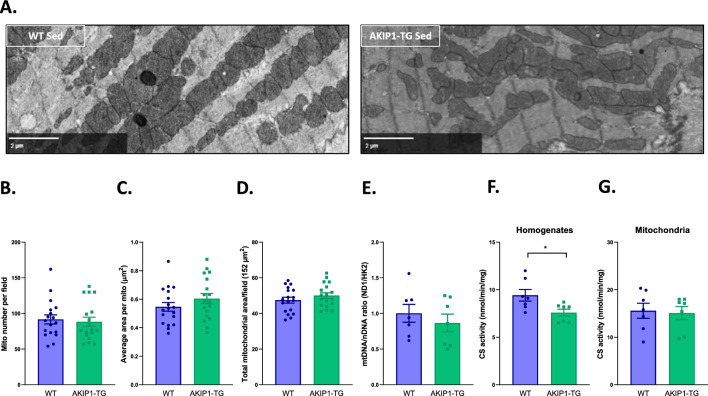
Mitochondrial morphology and mass: electron microscopy and mitochondrial content markers. Electron microscopy (EM) imaging to identify changes in mitochondrial morphology (N = six images of N = 3/group). Shown are (**A**) typical examples of EM images focused on the mitochondria. (**B**) Mitochondrial number per field, (**C**) average area per mitochondria in squared micrometres (µm^2^), and (**D**) total mitochondrial area per field of 152 µm^2^. Shown are (**E**) mitochondrial to nuclear DNA ratio (mtDNA/nDNA) (N = 7/group), (**F**) citrate synthase (CS) activity from homogenate samples in nanomole per minute per mg protein (nmol/min/mg) (N = 7/group) and (**G**) CS activity from isolated mitochondrial samples in nmol/min/mg (N = 7/group). Raw EM data with zoomable files at high resolution are accessible at www.nanotomy.org. ND1, NADH dehydrogenase 1; HK2, hexokinase 2; WT, wild type mice; AKIP1-TG, AKIP1 transgenic mice. Graphs represent mean ± standard error of the mean (SEM). Statistical analysis for comparing two groups was performed with Student’s *t-*test or Mann–Whitney U test. < 0.05 was considered statistically significant; p* < 0.05. Scale bars indicate 2 µm.

Since mitochondrial function in AKIP1-TG mice was lowered, we aimed to also assess whether mitochondrial morphology and architecture was altered using electron microscopy. The mitochondrial number per field as well as the average area per mitochondrion did not differ between WT and AKIP1-TG mice (Fig. 2A–C). Accordingly, the total mitochondrial area per field of 152 µm^2^ was also unaffected (Fig. 2D). In addition to these quantifications, the overall mitochondrial cristae structure and density did not appear to be altered. Together these findings suggest that mitochondrial morphology, architecture and abundance are not affected by AKIP1 overexpression.

The ratio of mitochondrial to nuclear DNA, an indirect measure of mitochondrial mass, remained unchanged between both genotypes (Fig. 2E). In contrast, citrate synthase activity in homogenates, another indirect measure of mitochondrial mass, was slightly, but significantly decreased in AKIP1-TG mice (Fig. 2F). This was also observed for CS mRNA levels in homogenates (Supplementary Fig. S4). Here it is usually implicitly assumed that CS activity per mitochondrial mass is constant, which was indeed the case (Fig. 2G). The CS data therefore suggest that mitochondrial content is indeed somewhat less in AKIP1-TG mice. However, taking into account together the EM, mtDNA and CS data, we concluded that overall, there was no change in abundance of mitochondria.

### The levels of mitochondrial proteins involved in energy metabolism are decreased in AKIP1-TG mice

**Figure 3:**
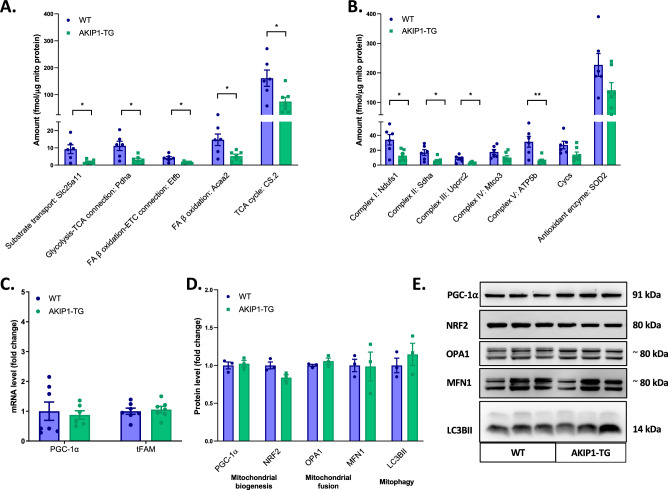
Mitochondrial proteome levels and mitochondrial dynamics: proteomics and molecular markers. In (**A**) and (**B**) quantification of a broad-spectrum mitochondrial proteins using mitochondrial-targeted proteomics is shown (N = 6–7/group). In (**A**) mitochondrial proteins are shown related to substrate transport (presented is Slc25a11 in femtomol per µg of total mitochondrial protein (fmol/µg mito protein)), the connection between glycolysis and the TCA cycle (presented is Pdha in fmol/µg mito protein), the connection between FA β-oxidation and the ETC (presented is Etfb in fmol/µg mito protein), fatty acid β-oxidation (presented is Acaa2 in fmol/µg mito protein), the TCA cycle (presented is CS in fmol/µg mito protein). In (**B**) mitochondrial proteins are shown related to the ETC and OXPHOS (presented are Ndufs1, Sdha, Uqcrc2, Mtco3, ATP5b and Cycs in fmol/µg mito protein) and related to the antioxidant enzymes (presented is SOD2 in fmol/µg mito protein). Molecular markers regarding mitochondrial dynamics are presented in the following panels. Shown are (**C**) mRNA expression of peroxisome proliferator-activated receptor gamma coactivator 1-alpha (PGC-1α) and mitochondrial transcription factor A (tFAM) (N = 7/group), (**D**) protein levels of PGC-1α, nuclear respiratory factor 2 (NRF2), optic atrophy 1 (OPA1), mitofusin 1 (Mfn1) and light chain 3B II (LC3BII) (N = 3/group), (**E**) this panel depicts typical examples of western blots, full western blots including the loading controls are attached in Supplementary Figs. S5–S9. WT, wild type mice; AKIP1-TG, AKIP1 transgenic mice. Graphs represent mean ± standard error of the mean (SEM). Statistical analysis for comparing two groups was performed with Student’s *t-*test or Mann–Whitney U test. < 0.05 was considered statistically significant; p* < 0.05, p** < 0.01.

To further unravel whether there were actual changes in levels of proteins in mitochondrial energy metabolism and related pathways, we performed targeted proteomics in cardiac isolated mitochondria samples. Indeed, a large group of proteins involved in substrate transport, pyruvate dehydrogenase, TCA cycle, fatty acid β-oxidation and OXPHOS were consistently lower in AKIP1-TG mice compared to WT mice (Fig. 3A, B). As briefly mentioned above, the antioxidant protein superoxide dismutase 2 (SOD2) was not significantly altered in AKIP1-TG mice (Fig. 3B). It is important to mention that the selected protein levels were normalized to mitochondrial protein. Since the mitochondrial mass was slightly decreased or unaltered (above), this implies that the levels of these proteins were also decreased per total cell protein. Altogether, these findings show that the quantified mitochondrial proteins involved in energy metabolism are decreased with AKIP1 overexpression. Full dataset, including a graphical representation, can be accessed in Supplementary Information File 2.

### AKIP1 overexpression does not alter mitochondrial dynamics

Subsequently, we assessed whether changes in mitochondrial protein levels were associated with changes in mitochondrial dynamics. First, we demonstrated that markers for mitochondrial biogenesis of peroxisome proliferator-activated receptor gamma coactivator 1-alpha (PGC-1α) and mitochondrial transcription factor A (tFAM) were not altered at mRNA level (Fig. 3C). In corroboration with this, protein levels of PGC-1α and NRF2 were similar in both WT and AKIP1-TG mice (Fig. 3D, E). Next, we assessed protein levels of mitochondrial fusion, including optic atrophy 1 (OPA1) and mitofusin 1 (MFN1) factors, which remained unchanged in AKIP1-TG mice (Fig. 3D, E). We also assessed light chain 3B II (LC3BII) as a marker for mitophagy, which was not altered with AKIP1 overexpression (Fig. 3D, E). Accordingly, the markers for mitochondrial biogenesis, mitochondrial fusion and mitophagy were all unaffected by cardiomyocyte-specific overexpression of AKIP1.

### Exercise decreases mitochondrial state 3 respiration for pyruvate but does not further decrease other mitochondrial functional parameters in AKIP1-TG mice

**Figure 4:**
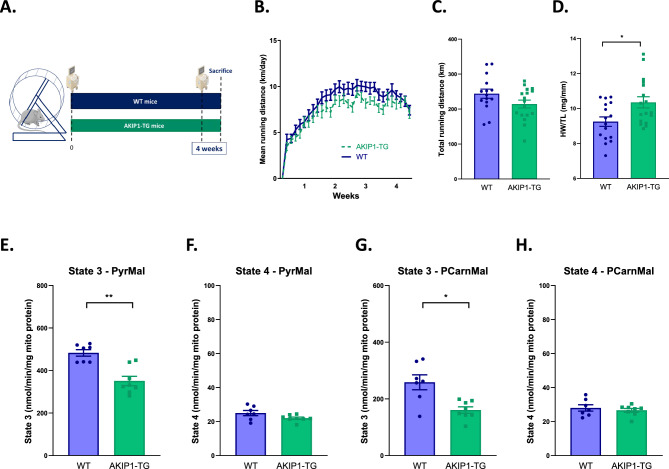
Cardiac and mitochondrial adaptation after exercise: cardiac weights and mitochondrial respiratory states. Shown are (**A**) graphical abstract depicting the experimental model, (**B**) mean running distance in kilometres per day (km/day) over a four-week period (N = 15–17/group), (**C**) cumulative total running distance in km after the four-week period (N = 15–17/group), (**D**) heart weight to tibia length ratio (HW/TL) in milligrams per millimetre (mg/mm) (N = 16–17/group), (**E**) state 3 respiration with pyruvate and malate (PyrMal) in nanomole per minute per milligram of mitochondrial protein (nmol/min/mg mito protein) (N = 7–8/group), (**F**) state 4 respiration for PyrMal in nmol/min/mg mito protein (N = 7–8/group), (**G**) state 3 respiration for palmitoyl carnitine and malate (PCarnMal) substrates in nmol/min/mg mito protein (N = 7–8/group), (**H**) state 4 respiration for PCarnMal in nmol/min/mg mito protein (N = 7–8/group). WT, wild type mice; AKIP1-TG, AKIP1 transgenic mice. Graphs represent mean ± standard error of the mean (SEM). Statistical analysis for comparing two groups was performed with Student’s *t-*test or Mann–Whitney U test. < 0.05 was considered statistically significant; p* < 0.05, p** < 0.01. Part of the illustration in panel A contains images from Servier Medical Art by Servier, licensed under a Creative Commons Attribution 3.0 unported license.

To assess the role of AKIP1 in the mitochondrial adaptation to exercise, we subjected WT and AKIP1-TG mice to four weeks of voluntary wheel running. Similar to previous studies^7^, AKIP1-TG and WT mice exhibited similar exercise performance, yet exercise-induced cardiac hypertrophy was significantly augmented in AKIP1-TG mice (Fig. 4A–D).

After four weeks of voluntary exercise, mitochondrial state 3 respiration on a glucose-derived substrate (pyruvate) was significantly diminished in AKIP1-TG mice compared to WT mice (Fig. 4E), whereas respiratory state 4 remained unchanged (Fig. 4F). Mitochondrial state 3 respiration on a fatty acid-derived substrate (palmitoyl carnitine) was also less in the AKIP1-TG group (Fig. 4G), but oxygen consumption rates were similar to what was observed in sedentary mice. State 4 respiration on palmitoyl carnitine remained unaltered (Fig. 4H). Using targeted proteomics, mitochondrial protein levels involved in energy metabolism were determined and the differences observed after 4 weeks of exercise were also similar to the changes observed in sedentary mice (Supplementary Information File 2).

### AKIP1 overexpression promotes mitochondrial fission and mitochondrial turnover after exercise

**Figure 5:**
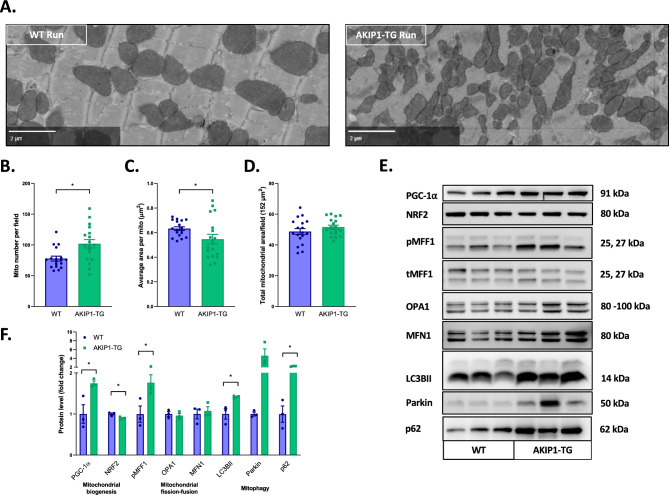
Mitochondrial morphology and dynamics: regulation of mitochondrial fission and mitophagy. Electron microscopy (EM) imaging to identify changes in mitochondrial morphology (N = six images of N = 3/group). Shown are (**A**) typical examples of EM images focused on the mitochondria, (**B**) mitochondrial number per field, (**C**) average area per mitochondria in squared micrometres (µm^2^), and in (**D**) total mitochondrial area per field of 152 µm^2^. Molecular markers for mitochondrial dynamics including mitochondrial biogenesis, fission and fusion and mitophagy. Shown are (**E**) typical examples of western blots, full western blots including the loading controls are attached in Supplementary Figs. S5–S12 and in (**F**) a panel of protein levels including peroxisome proliferator-activated receptor gamma coactivator 1-alpha (PGC-1α), nuclear respiratory factor 2 (NRF2), phosphorylation of mitochondrial fission factor 1 (pMFF1), optic atrophy 1 (OPA1), mitofusin 1 (Mfn1), light chain 3B II (LC3BII), Parkin and ubiquitin protein 62 (p62) (N = 3/group). Raw EM data with zoomable files at high resolution are accessible at www.nanotomy.org. WT, wild type mice; AKIP1-TG, AKIP1 transgenic mice. Graphs represent mean ± standard error of the mean (SEM). Statistical analysis for comparing two groups was performed with Student’s *t-*test or Mann–Whitney U test. < 0.05 was considered statistically significant; p* < 0.05.

Exercise is known to promote mitochondrial adaptations in cardiac tissue, including changes in mitochondrial biogenesis, fission and fusion, and mitophagy^1^. We next assessed whether exercise-induced changes in mitochondrial morphology and dynamics would be influenced by AKIP1 overexpression. In contrast to our observations in sedentary mice, the mitochondrial number per field was significantly increased in AKIP1-TG mice (Fig. 5A, B), accompanied by a significant reduction in the average mitochondrial size (Fig. 5C). The total mitochondrial area was however not influenced by AKIP1 (5D). With regards to mitochondrial architecture, the organisation of interfibrillar mitochondria was disorganized throughout cardiac tissue of AKIP1-TG mice, which was not observed in EM scans of sedentary mice. These findings suggest that AKIP1 affects changes in mitochondrial morphology and architecture in response to exercise.

**Figure 6:**
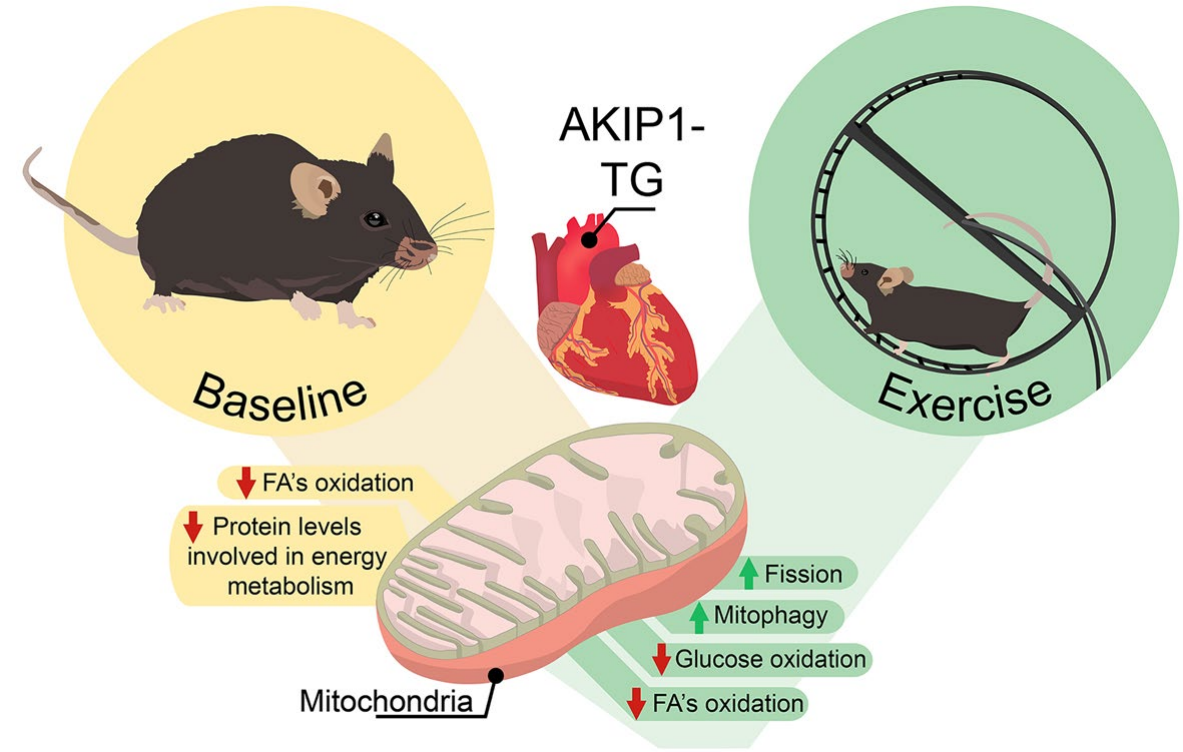
Graphical abstract: mitochondrial alterations with AKIP1 overexpression. Baseline mitochondrial changes in AKIP1-TG mice are depicted, included a reduction in mitochondrial respiratory capacity for fatty acids and decrements in protein levels of mitochondrial proteins involved in energy metabolism. Upon exercise, a subsequent set of mitochondrial changes occur in AKIP1-TG mice. As in sedentary mice, fatty acid oxidation is decreased, but now also respiratory capacity for glucose substrates is decreased. These changes were accompanied by association with changes in markers for mitochondrial fission and mitophagy, suggesting enhanced mitochondrial turnover with exercise and AKIP1 overexpression. AKIP1-TG, AKIP1 transgenic mice; FA’s, fatty acids.

We subsequently performed an in-depth analysis of markers for mitochondrial dynamics including mitochondrial biogenesis, fission and fusion, as well as mitophagy. First we assessed protein levels of markers for mitochondrial biogenesis. PGC-1α levels were significantly increased, whereas NRF2 levels were slightly lowered in AKIP1-TG mice. These contrasting results may suggest that mitochondrial biogenesis is dysregulated with AKIP1 and exercise (Fig. 5E, F), Next, we focused on mitochondrial dynamics. The phosphorylation of mitochondrial fission factor 1 (pMFF1) was significantly increased in AKIP1-TG mice after exercise (Fig. 5E, F), suggesting that pathways stimulating mitochondrial fragmentation were augmented. Mitochondrial fusion however did not appear to be altered as levels of OPA1 and Mfn1 were unaltered (Fig. 5E, F). In line with increased mitochondrial fragmentation, markers for mitophagy and autophagy including LC3BII, Parkin and p62, were increased in AKIP1-TG mice after exercise (Fig. 5E, F). The results of this study are summarized in Fig. 6.

## Discussion

We aimed to investigate the role of cardiomyocyte-specific overexpression of AKIP1 on mitochondrial function and the mitochondrial response to exercise. Mice with AKIP1 overexpression displayed decreased mitochondrial state 3 respiratory capacity for a fatty-acid derived substrate (palmitoyl carnitine), which was also observed during maximal respiration after addition of FCCP. However, mitochondria did not show mitochondrial DNA damage or enhanced expression of oxidative stress markers. Mitochondrial morphology remained unaltered with AKIP1 overexpression, whereas mitochondrial protein levels involved in energy metabolism were significantly lowered in AKIP1-TG mice; this was however without changes in mitochondrial dynamics. In response to exercise, a surprising reduction in state 3 respiration on carbohydrates (pyruvate) was observed in AKIP1-TG mice, which was associated with the augmentation of mitochondrial turnover (depicted in graphical abstract Fig. 6). Together, our findings suggest that AKIP1 regulates mitochondrial protein levels involved in energy metabolism and promotes mitochondrial turnover after exercise.

A study using NRVM showed that AKIP1 overexpression improved mitochondrial respiration and increased subsequent ATP production^10^. In corroboration with this, mitochondrial ROS production was also decreased^10^. Together, this study suggested that AKIP1 regulates mitochondrial function in a positive manner. In subcellular fraction data, AKIP1 also localized to the mitochondria^8^. Additionally, AKIP1 overexpression provided protection to ischemia–reperfusion injury through mitochondrial changes in two individual studies^8,9^. Contrarily to the findings of in vitro data and our hypothesis that AKIP1 could improve mitochondrial function, we observed reductions in mitochondrial state 3 respiration for a fatty-acid derived substrate (palmitoyl carnitine) in sedentary mice with AKIP1 overexpression. Interestingly, in view of the proteomics data, in which PDH and complex 1 were lowered, this reduction was not observed in state 3 respiration of a glucose-derived substrate (pyruvate). Whether this is a metabolic shift in substrate utilization is unclear based on our current data, but we can hypothesize that substrate utilization could potentially be shifting towards more glucose utilization**.** With exercise however, state 3 respiration was diminished for both substrates, suggesting an overall reduction in respiratory capacity irrespective of the substrate, which is not indicative for a beneficial adaptation^4^. It is of importance to consider that the findings from our proteomics data, in particular the reductions observed in several metabolic pathways, must be compensated by increments in other mitochondrial proteins since there was an input of equal quantities of mitochondrial protein. This suggests that proteins involved in other mitochondrial processes are potentially increased, which may also contribute to the overall effects observed. While our results may appear to be contradictory to our previous in vitro study, it is important to note that assays were performed in neonatal cardiomyocytes, which are more reliant on glucose metabolism. The results may have been different if we had studied adult mature cardiomyocytes.

Physiological cardiac hypertrophy can develop in response to physiological stimuli such as exercise and pregnancy, and entails beneficial cardiac growth often mediated by the IGF1-Akt-C/EBPβ-CITED4 pathway^1–3,18^. Physiological cardiac hypertrophy induced by exercise is however associated with more than cardiomyocyte growth and is accompanied by for example an expansion of the capillary network^19^ and beneficial mitochondrial adaptations^1–3,18^. Previous in vitro studies have determined that AKIP1 promotes physiological cardiomyocyte growth through Akt signalling^6^. Also, our recent exercise study, has determined the essential role of AKIP1 for physiological cardiomyocyte elongation regulated by the SRF signalosome and physiological cardiac remodelling via the Akt-C/EBPβ-CITED4 pathway^7^. Together, these findings suggest that AKIP1 regulates the physiological cardiac growth response and serves as a potential therapeutic target to induce physiological growth in a pathological setting such as heart failure. In this study we aimed to identify if AKIP1 also affected other aspects of physiological cardiac hypertrophy, specifically the mitochondrial adaptation to exercise. In contrast to our hypothesis, we observed reductions in mitochondrial function. However, we also observed an increase in mitochondrial turnover in response to exercise with AKIP1 overexpression. In the literature, there are several discrepancies with regards to mitochondrial dynamics and mitophagy^1^. However, several studies describe the necessity for mitochondrial fission and mitophagy^20^ as a beneficial adaptation to exercise in health and disease settings^21–24^. Whether the AKIP1-induced mitochondrial turnover with exercise is beneficial and required to enable a physiological hypertrophic growth response, remains to be further explored. Of note, several lines of evidence in our study indicate that mitochondria do not appear to be damaged. Moreover, we did not detect evidence for mitochondrial oxidative stress or fibrosis, indicating that the mitochondrial changes induced by AKIP1 were not necessarily maladaptive.

In our study we investigated the role of A Kinase Interacting Protein 1 (AKIP1) on mitochondrial function and homeostasis. Our findings are in line with several recent publications on this topic, including a book chapter by Sorriento et al.^25^, which highlights the different mechanistic functions of kinases on mitochondrial function, biogenesis, fission and fusion and mitophagy. For instance, protein kinase A (PKA) and glycogen synthase kinase 3-beta (GSK3 *β*) induce phosphorylation of multiple (mitochondrial) proteins and promote their translocation into the mitochondria^25^. It has also been shown that the activation of the mitochondrial NO-cGMP-protein kinase G pathway regulated cytosolic calcium-induced apoptosis in cardiomyocytes^26^. Also, the inhibition of G-protein-coupled receptor kinase 2 (GRK2) showed to play an important beneficial role in cardiac mitochondrial metabolism in a heart failure setting^27^. Finally, the mitochondrial derived peptide humanin also appears to regulate mitochondrial pathways and to reduce oxidative stress in cardiovascular aging^28^. Together, these studies suggest that protein kinases are indeed critical for mitochondrial function and homeostasis.

### Strengths and limitations

In this study, we have applied a diverse set of techniques to perform broad phenotyping of mitochondria. This allowed for the assessment of multiple aspects of mitochondrial homeostasis, and we were able to analyse the fundamental processes of mitochondria. The complementary nature of these techniques may be considered as one of the strengths of this work. Our study, however, also includes several limitations that could potentially also cause forms of bias. First, we used an experimental model of voluntary wheel running. Despite the fact that the average amount of exercise performed was similar in WT and AKIP1-TG mice, we were unable to control the exact amount of exercise performed by individual mice. Using a method of forced endurance exercise would have allowed us to control for this more thoroughly. Some degree of selection bias is inferred by the fact that only male mice were used in this study, as well as that the age range of the experiment was set for 8–12 weeks old, which is relatively young for adult mice. Another limitation of this study is that we do not report ROS production or downstream effects of oxidative damage. Additionally, it is of importance to consider that some of the mitochondrial processes assessed in this study are dynamic and short-lived, including mitochondrial fission and mitophagy. The fact that we only assessed these pathways after four weeks does not allow us to draw conclusions about other timepoints.

### Future perspectives

It remains unknown via which mechanism AKIP1 affects the mitochondrial proteome and the mitochondrial alterations in the exercise groups. The specific cell signalling pathways involved and whether AKIP1 interacts with these directly or indirectly remains unanswered. A potential regulator is AMPK and its localization to the mitochondria, which has recently been discovered in skeletal and cardiac muscle as a regulator of mitochondrial quality control^22^. Also downstream Ulk activation has been shown to be essential for exercise-induced mitochondrial turnover^29^. Further studies are therefore required to increase the knowledge on the underlying molecular mechanisms of the regulatory pathways for the findings of this study. Additionally, it should be further explored whether mitochondrial turnover in AKIP1-TG mice after exercise is adaptive in nature, potentially acting as a predisposition for the development of physiological cardiac hypertrophy.

Adaptive changes in response to exercise are highly dependent upon the type of exercise, ranging from acute to chronic- and endurance to resistance exercise training^1^. In this study we employed an experimental model of voluntary wheel running in mice, which resembles recreative endurance exercise. Current literature describes multiple other types of exercise protocols in animal models that may be used when studying exercise adaptations^30^. These models include resemblance with resistance, more acute and intense exercise training. Exploring the effect of AKIP1 with different exercise regimens may give rise to results approaching the effects of exercise from another perspective.

## Conclusion

Taken together, the findings of this study indicate that AKIP1 regulates mitochondrial protein levels involved in energy metabolism and promotes mitochondrial turnover after exercise. The underlying molecular mechanisms of the mitochondrial changes should be further explored, and future studies should reveal whether these findings are essential for AKIP1-induced physiological cardiac hypertrophy.

### Supplementary Information

Supplementary Information 1.

Supplementary Information 2.

## Data Availability

The data from the current study are available from the corresponding author on reasonable request.
