# Supplementary material for: A Kinase Interacting Protein 1 regulates mitochondrial protein levels in energy metabolism and promotes mitochondrial turnover after exercise
Source: Sci Rep. 2023 Nov 1;13:18822. doi: 10.1038/s41598-023-45961-z (PMC10620178; doi:10.1038/s41598-023-45961-z)
Supplement: Supplementary file 1 — Supplementary Information 1. [file 41598_2023_45961_MOESM1_ESM.pdf]

Supplementary information

A Kinase Interacting Protein 1 regulates mitochondrial protein levels in energy metabolism and promotes mitochondrial turnover after exercise

Kirsten T. Nijholt, BSc[1], Pablo I. Sánchez-Aguilera, MSc[1], Belend Mahmoud, MD[1], Albert Gerding, BSc[2], Justina C. Wolters, PhD[3], Anouk H.G. Wolters, BSc[4], Ben N. G. Giepmans, PhD[4], Herman H.W. Silljé, PhD[1], Rudolf A. de Boer, MD, PhD[1,5], Barbara M. Bakker, PhD[2], B. Daan Westenbrink, MD, PhD[1*]

[1]Department of Cardiology, University Medical Centre Groningen, University of Groningen, Groningen, The Netherlands

[2]Department of Metabolic Disease, University Medical Centre Groningen, University of Groningen, Groningen, The Netherlands

[3]Department of Pediatrics, Systems Medicine of Metabolism and Signalling, University Medical Centre Groningen, University of Groningen, Groningen, The Netherlands

[4]Department of Biomedical Sciences of Cells and Systems, University Medical Centre Groningen, University of Groningen, Groningen, The Netherlands

[5]Department of Cardiology, Erasmus University Medical, Rotterdam, The Netherlands

*Corresponding author:
B.D. Westenbrink, MD, PhD
Department of Cardiology, University Medical Centre Groningen
Hanzeplein 1, 9713 GZ Groningen, The Netherlands
P.O. Box 30.001, 9700 RB Groningen, The Netherlands
Phone: +31 50 361 23 55, fax: +31 50 361 13 47, email b.d.westenbrink@umcg.nl

Short title: AKIP1 controls the mitochondrial metabolic proteome

Tables

Supplementary Table S1. Primer sequences for quantitative real-time polymerase chain reaction (qRT-PCR)

	**5'-3' Forward**	**5'-3' Reverse**
36B4	AAGCGCGTCCTGGCATTGTC	GCAGCCGCAAATGCAGATGG
AKIP1	AGAGGCGAGACATCTGTTGG	TAGGTCCCTGGGTAGACTTC
mtDNA-long	AAAATCCCCGCAAACAATGACCACCC	GGCAATTAAGAGTGGGATGGAGCCAA
mtDNA-short	CCTCCCATTCATTATCGCCGCCCTTGC	GTCTGGGTCTCCTAGTAGGTCTGGGAA
NRF2	ATGGACTTGGAGTTGCCACC	TCTTGCCTCCAAAGGATGTCA
NOX2	CTCGACAAGGATTCGAAGAC	GTGCTATCATCCAAGCTACC
ND1	CTAGCAGAAACAAACCGGGC	CCGGCTGCGTATTCTACGTT
HK2	GCCAGCCTCTCCTGATTTTAGTGT	GGGAACACAAAAGACCTCTTCTGG
CS	CTGTGGTCTTCCTGGTCGTT	TCATTCCTGAGCCCTTGTTC
PGC-1α	CGGAAATCATATCCAACCAG	TGAGGACCGCTAGCAAGTTTG
tFAM	AAAGGATGATTCGGCTCAGG	ACTTCGACGGATGAGATCAC

Table S1. Primer sequences for the following genes: *(1)* 36B4, housekeeping gene; *(2)* AKIP1, a kinase interacting protein 1; *(3)* mtDNA-long, mitochondrial DNA long fragment; *(4)* mtDNA-short, mitochondrial DNA short fragment; *(5)* NRF2, nuclear respiratory factor 2; *(6)* NOX2, NADPH oxidase 2; *(7)* ND1, NADH dehydrogenase 1; *(8)* HK2, hexokinase 2; *(9)* CS, citrate synthase *(10)* PGC-1α, peroxisome proliferator-activated receptor gamma coactivator 1-alpha; *(11)* tFAM, mitochondrial transcription factor A.

Supplementary Table S2. List of antibodies used for Western blot.

	Primary antibody
GAPDH	10R-G109A, Fitzgerald, USA
Total protein stain	Revert total protein stain, LI-COR, USA
PGC-1α	ab54481, Abcam, United Kingdom
NRF2	ab31163, Abcam, United Kingdom
OPA1	80471, Cell Signaling, USA
MFN1	14739, Cell Signaling, USA
LC3BII	2775, Cell Signaling, USA
pMFF1	49281, Cell Signaling, USA
tMFF1	86668, Cell Signaling, USA
Parkin	4211, Cell Signaling, USA
P62	ab56416, Abcam, United Kingdom

Table S2. Antibodies used for the following proteins: *(1)* GAPDH, glyceraldehyde 3-phosphate dehydrogenase; *(2)* total protein stain, *(3)* PGC-1α, peroxisome proliferator-activated receptor gamma coactivator 1-alpha; *(4)* NRF2, nuclear respiratory factor 2; *(5)* OPA1, optic atrophy 1; *(6)* MFN1, mitofusin 1; *(7)* LC3BII, light chain 3B II; *(8)* pMFF1, phosphorylated mitochondrial fission factor 1; *(9)* total mitochondrial fission factor 1; *(10)* Parkin; *(11)* P62, ubiquitin protein 62.

Figures

Supplementary Figure S1. AKIP1 mRNA expression in WT and AKIP1-TG mice.

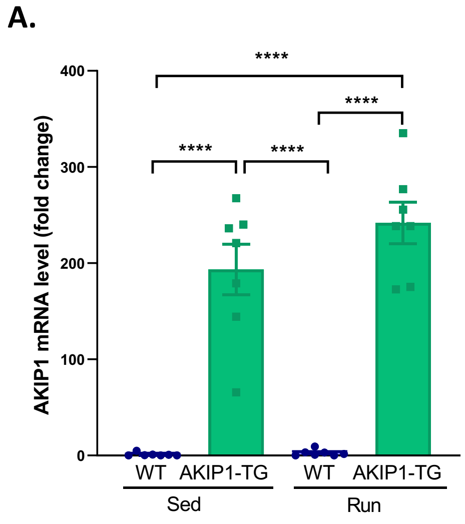

Figure S1. Shown are, *(A.)* mRNA expression of AKIP1 in all four groups (N=7/group). WT= wild type mice, AKIP1-TG= AKIP1 transgenic mice, Sed= sedentary, Run= running. Graphs represent mean ± standard error of the mean (SEM). Statistical analysis for comparing multiple groups was performed with Two-way ANOVA with post-hoc *Tukey* test. <0.05 was considered statistically significant; p****<0.001.

Supplementary Figure S2. Mitochondrial function: typical examples of respiration curves.

A.

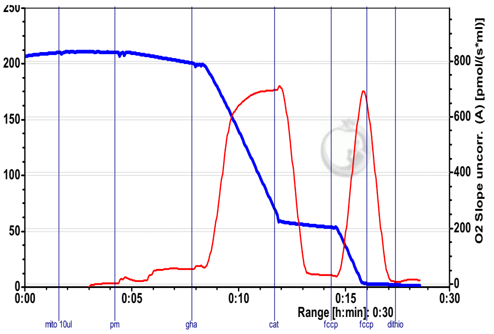

B.

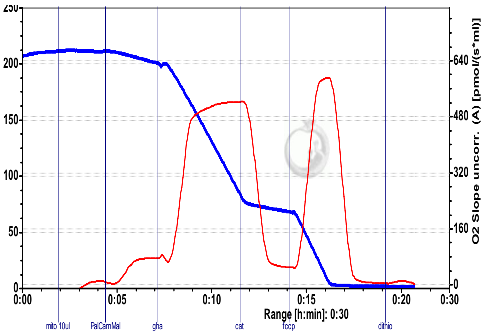

Figure S2. Shown are typical examples of Oroboros respiratory curve for *(A.)* pyruvate and malate (PyrMal) and *(B.)* palmitoyl carnitine and malate (PCarnMal) substrates.

Supplementary Figure S3. Mitochondrial integrity: markers for mitochondrial DNA damage and oxidative stress.

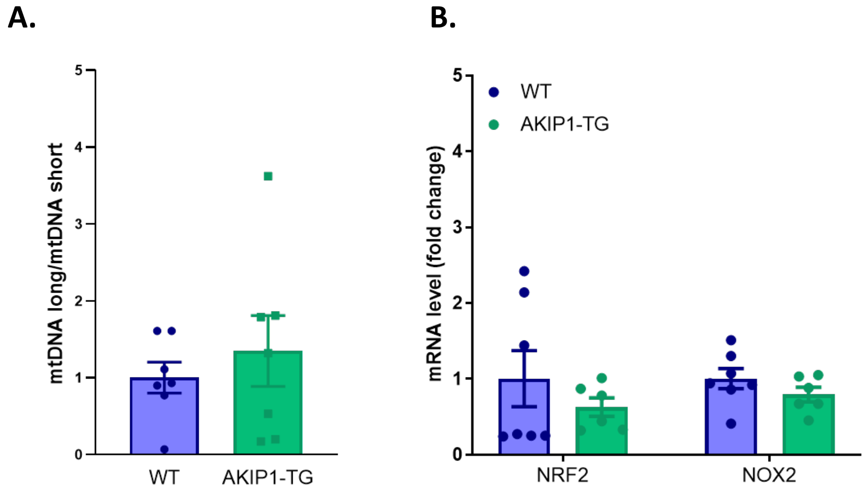

Figure S3. *(A.)* ratio of mitochondrial DNA long fragment (mtDNA long) to mitochondrial DNA short fragment (mtDNA short), as a measure for mitochondrial damage (N=7/group), *(B.)* mRNA levels for nuclear respiratory factor 2 (NRF2) and NADPH oxidase 2 (NOX2) (N=6-7/group). WT= wild type mice, AKIP1-TG= AKIP1 transgenic mice, Sed= sedentary, Run= running. Graphs represent mean ± standard error of the mean (SEM).

Supplementary Figures S4. Citrate synthase mRNA expression in homogenate samples.

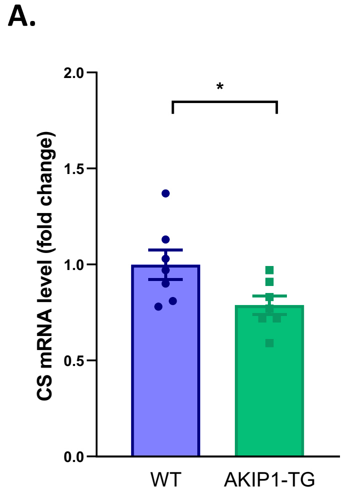

Supplementary Figure S4. *(A.)* mRNA expression of CS (N=7/group). CS= citrate synthase, WT= wild type mice, AKIP1-TG= AKIP1 transgenic mice, Sed= sedentary, Run= running. Graphs represent mean ± standard error of the mean (SEM). <0.05 was considered statistically significant; p*<0.05.

Supplementary Figure S5.

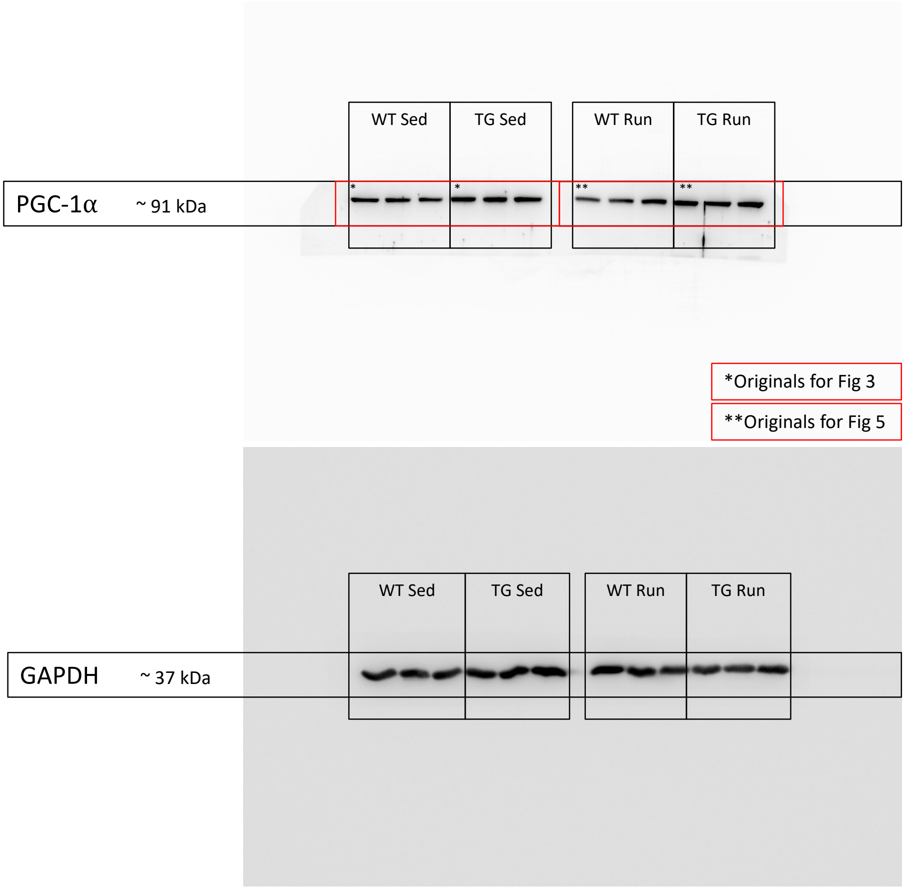

Supplementary Figure S5. Full western blot of PGC-1α, and housekeeping of GAPDH. PGC-1α= peroxisome proliferator-activated receptor gamma coactivator 1-alpha, GAPDH= glyceraldehyde 3-phosphate dehydrogenase, Sed= Sedentary, Run= Running, WT= wild type mice, TG= AKIP1 transgenic mice, Fig= Figure.

Supplementary Figures S6.

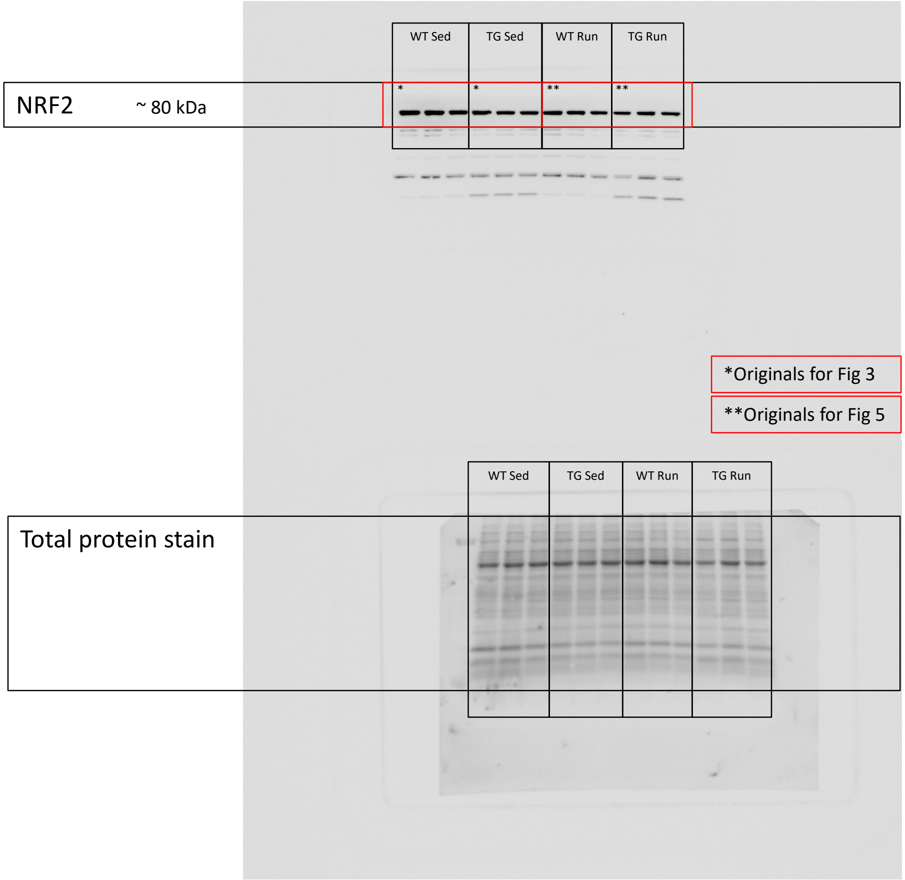

Supplementary Figure S6. Full western blot of NRF2 and housekeeping of GAPDH. NRF2= nuclear respiratory factor 2, GAPDH= glyceraldehyde 3-phosphate dehydrogenase, Sed= Sedentary, Run= Running, WT= wild type mice, TG= AKIP1 transgenic mice, Fig= Figure.

Supplementary Figures S7.

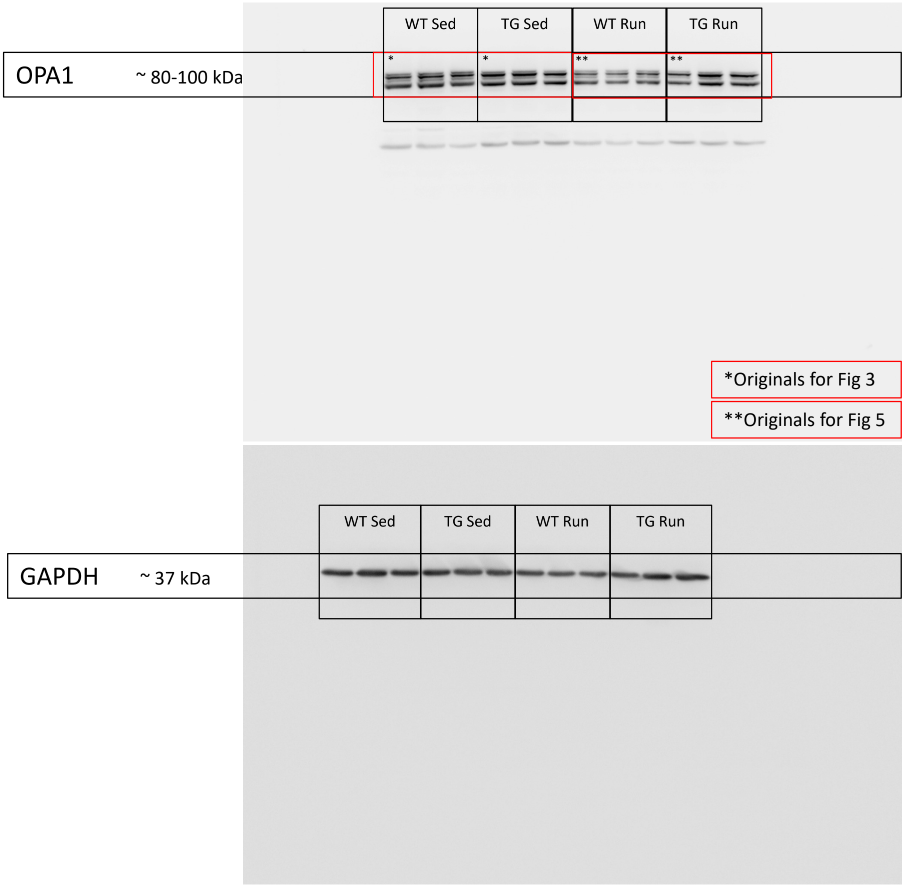

Supplementary Figure S7. Full western blot of OPA1 and housekeeping of GAPDH. OPA1= optic atrophy 1, GAPDH= glyceraldehyde 3-phosphate dehydrogenase, Sed= Sedentary, Run= Running, WT= wild type mice, TG= AKIP1 transgenic mice, Fig= Figure.

Supplementary Figures S8.

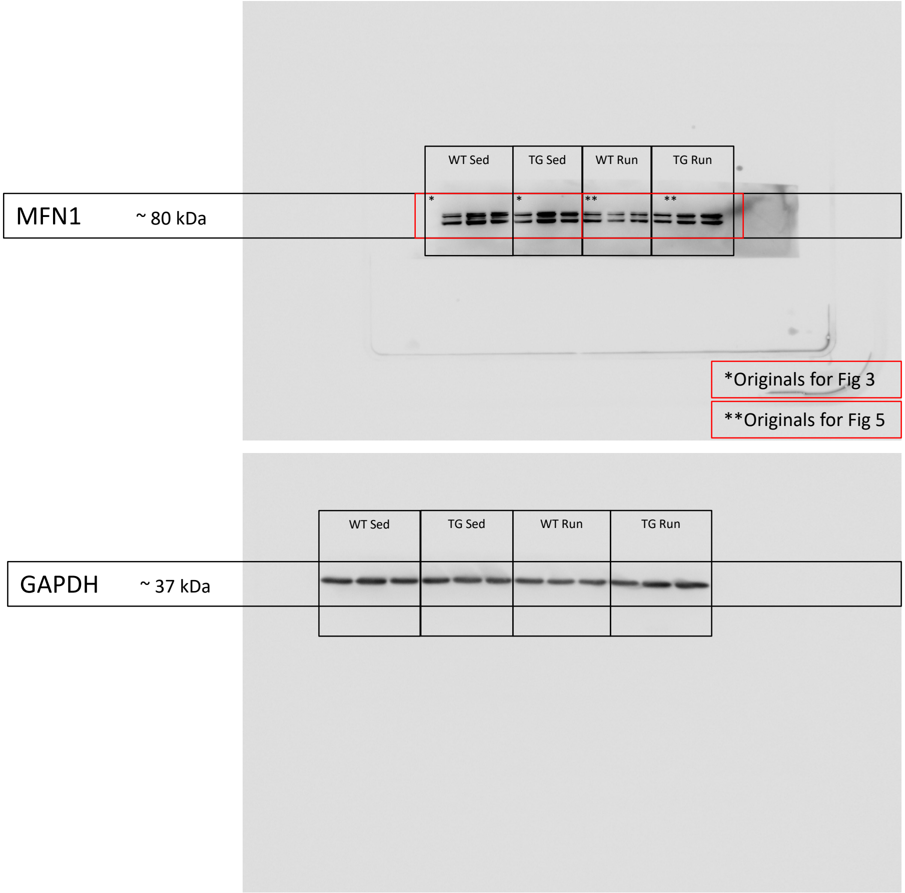

Supplementary Figure S8. Full western blot of MFN1 and housekeeping of GAPDH. MFN1= mitofusin 1, GAPDH= glyceraldehyde 3-phosphate dehydrogenase, Sed= Sedentary, Run= Running, WT= wild type mice, TG= AKIP1 transgenic mice, Fig= Figure.

Supplementary Figures S9.

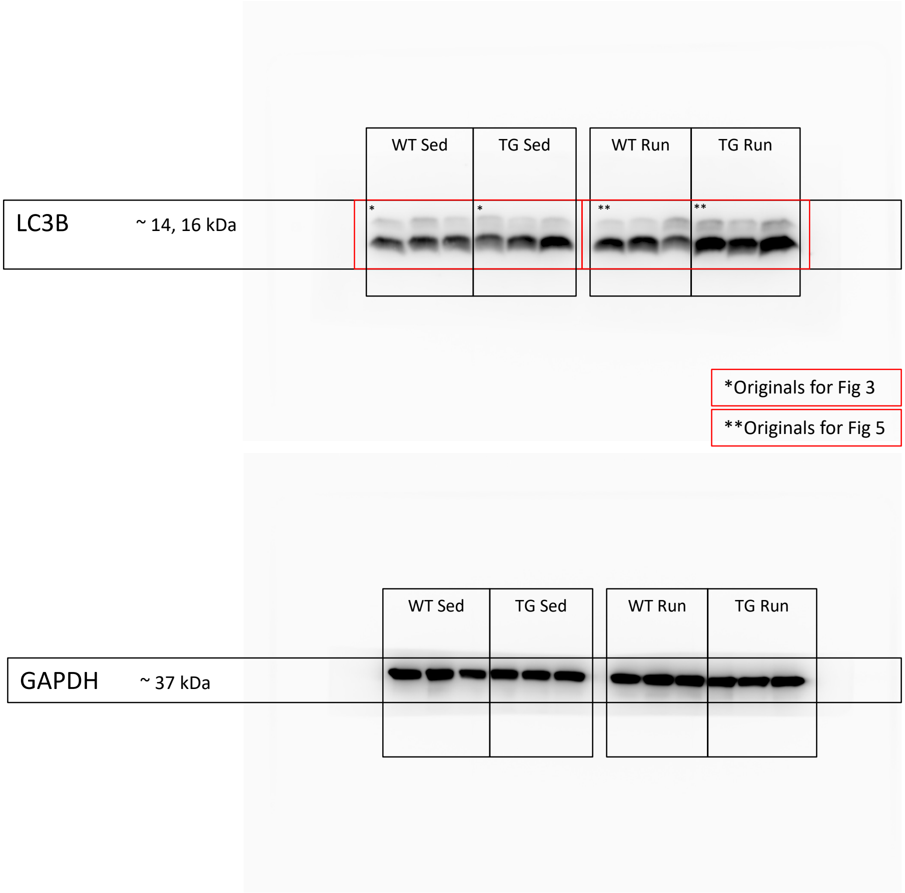

Supplementary Figure S9. Full western blot of LC3BII and housekeeping of GAPDH. LC3BII= light chain 3 B II, GAPDH= glyceraldehyde 3-phosphate dehydrogenase, Sed= Sedentary, Run= Running, WT= wild type mice, TG= AKIP1 transgenic mice, Fig= Figure.

Supplementary Figures S10.

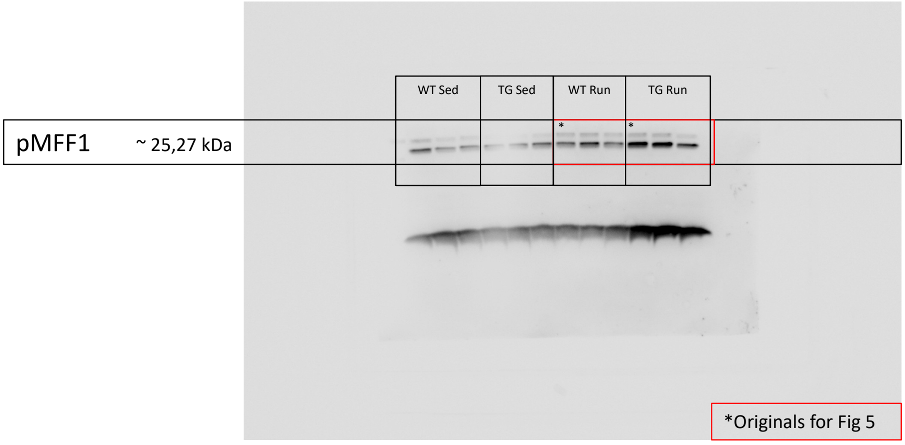

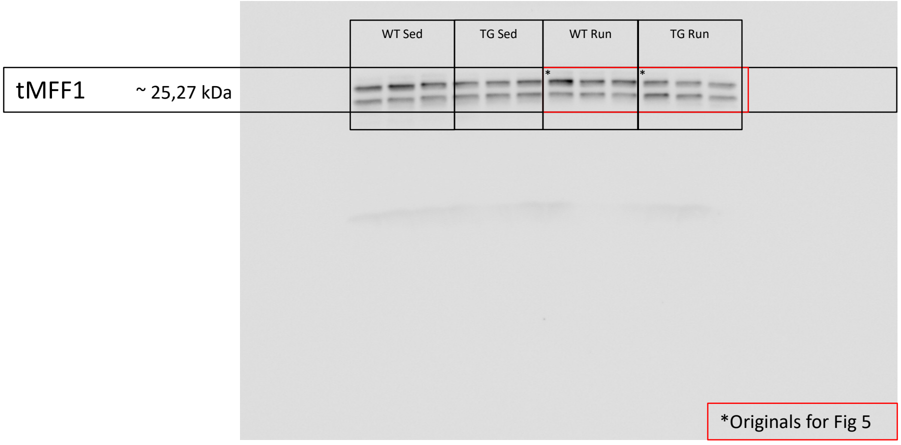

Supplementary Figure S10. Full western blot of pMFF1 and tMFF1. pMFF1= phosphorylated mitochondrial fission factor 1, tMFF1= total mitochondrial fission factor 1, Sed= Sedentary, Run= Running, WT= wild type mice, TG= AKIP1 transgenic mice, Fig= Figure.

Supplementary Figures S11.

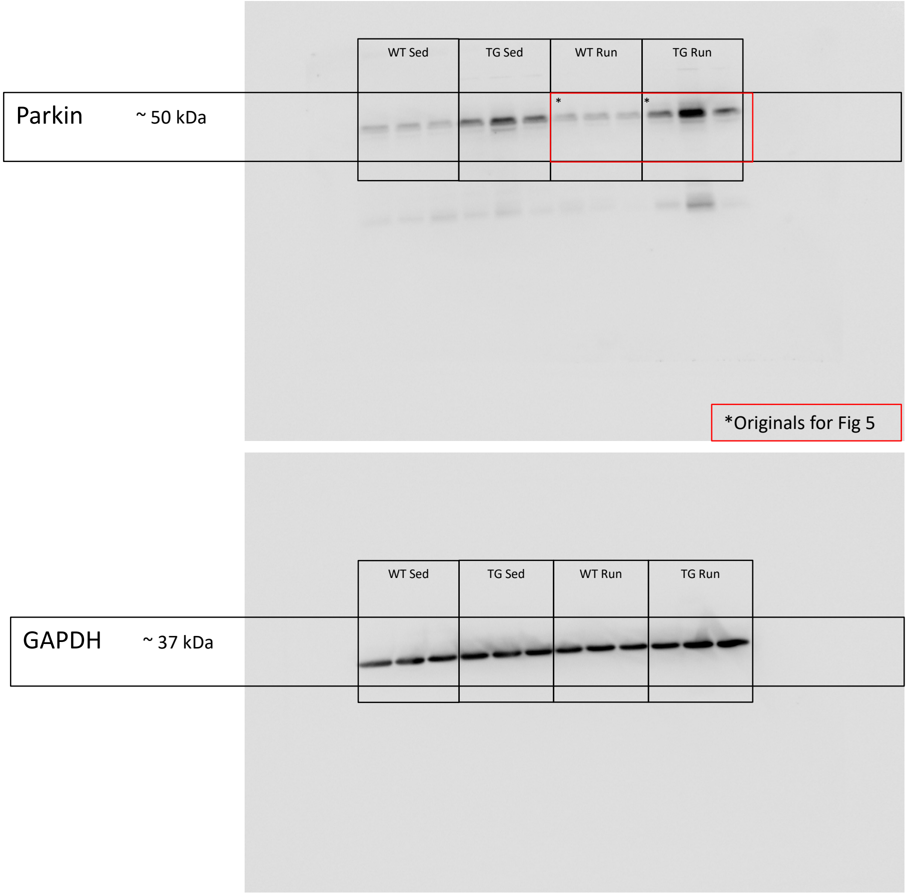

Supplementary Figure S11. Full western blot of Parkin and housekeeping of GAPDH. GAPDH= glyceraldehyde 3-phosphate dehydrogenase, Sed= Sedentary, Run= Running, WT= wild type mice, TG= AKIP1 transgenic mice, Fig= Figure.

Supplementary Figures S12.

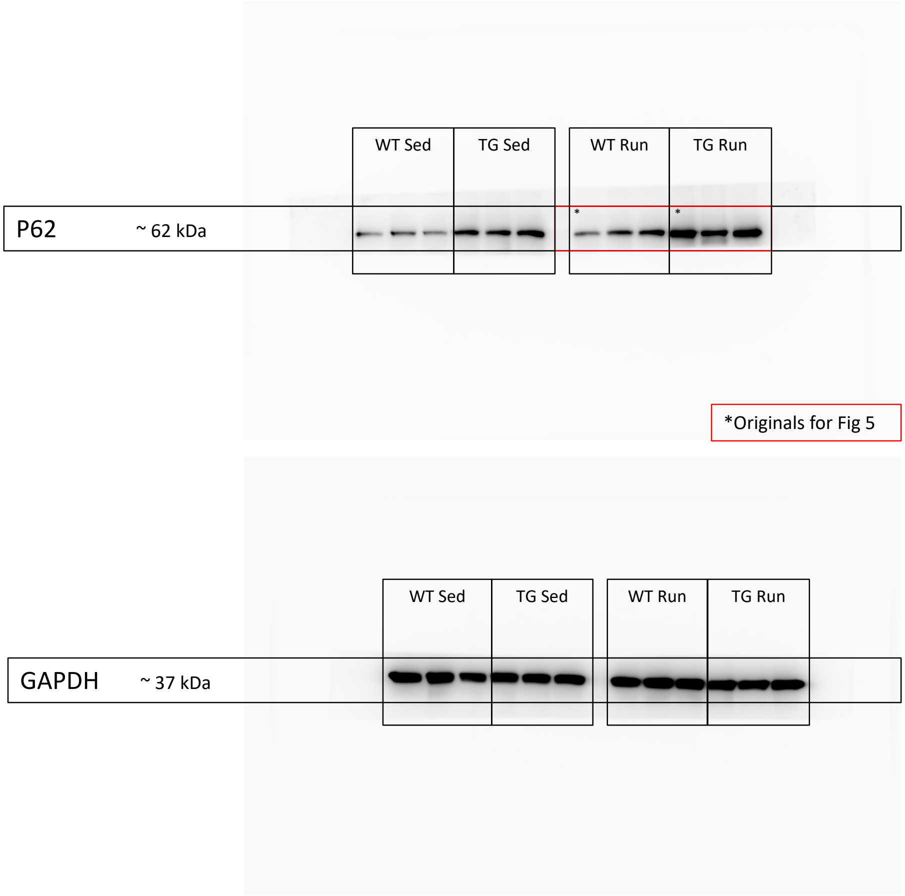

Supplementary Figure S12. Full western blot of p62 and housekeeping of GAPDH. P62= ubiquitin protein 62, GAPDH= glyceraldehyde 3-phosphate dehydrogenase, Sed= Sedentary, Run= Running, WT= wild type mice, TG= AKIP1 transgenic mice, Fig= Figure.
